# Clinical manifestations, prognosis, and treat-to-target assessment of pediatric lupus nephritis

**DOI:** 10.1007/s00467-021-05164-y

**Published:** 2021-08-11

**Authors:** Shiyuan Qiu, Hengci Zhang, Sijie Yu, Qin Yang, Gaofu Zhang, Haiping Yang, Qiu Li, Mo Wang

**Affiliations:** 1 Pediatric Internal Medicine Department, Chongqing You You Baobei Women’s and Children’s Hospital, Chongqing, China; 2 Guizhou Medical University, Guiyang, China; 3 Department of Nephrology, Ministry of Education Key Laboratory of Child Development and Disorders, National Clinical Research Center for Child Health and Disorders, China International Science and Technology Cooperation Base of Child Development and Critical Disorders, Children’s Hospital of Chongqing Medical University, Chongqing Key Laboratory of Pediatrics, Chongqing, China

**Keywords:** Systemic lupus erythematosus, Lupus nephritis, Children, Prognosis

## Abstract

**Background:**

Pediatric lupus nephritis (pLN) is one of the most refractory secondary kidney diseases in childhood. The treat-to-target (T2T) strategy has become the standard treatment for systemic lupus erythematosus (SLE). This study reviewed clinical features, overall remission status, and factors affecting prognosis, to guide pLN management according to T2T strategy.

**Methods:**

This single-center retrospective study studied 220 children diagnosed with LN from January 2012 to December 2018, with > 6-month follow-up data on 173 and complete data on 137 patients. Primary outcome was treatment failure (deterioration or no response) at the latest follow-up.

**Results:**

The most common pLN manifestation was proteinuria (81.36%). Females presented more often with rash (P<0.001) and alopecia (P=0.026) than males. Class IV LN (33.33%) was the most common grade on kidney biopsy. Median follow-up was 27.20 months (IQR, 15.78–44.45 months). One-, 3-, and 5-year cumulative overall survival rates were 93.5%, 87.8%, and 86.5%, respectively. The 5-year cumulative kidney survival rate was 97.1%. Regarding initial therapy, efficacy of corticosteroids combined with immunosuppressive agents was significantly better than corticosteroids alone (P=0.010). Factors with P<0.05 in univariate analysis, including hypoalbuminemia, higher SCr at diagnosis, lower eGFR at diagnosis, anti-dsDNA positivity, heavy proteinuria, hypertension, nervous-system involvement, treatment non-compliance, and SLEDAI-2K score, were used for logistic regression analysis. Logistic regression analysis showed hypertension (OR=0.845, P=0.011), nervous-system involvement (OR=4.240, P=0.005), treatment non-compliance (OR=6.433, P=0.001), and lower estimated glomerular filtration rate at diagnosis (OR=1.020, P=0.021) affected prognosis. At end of follow-up, 34.31% achieved varying levels of remission, and 8.76% were in low disease activity state (LDAS).

**Conclusions:**

pLN usually presented with proteinuria, and class IV LN was the dominant pathology. Hypertension, nervous-system involvement, treatment non-compliance, and lower eGFR at diagnosis were independent risk factors for poor prognosis of kidney outcomes. Compared with renal remission rate and cumulative overall survival rate, the proportion of targets achieved was not ideal, suggesting T2T strategy should be used to guide pLN management.

**Graphical abstract:**

**Figure:**
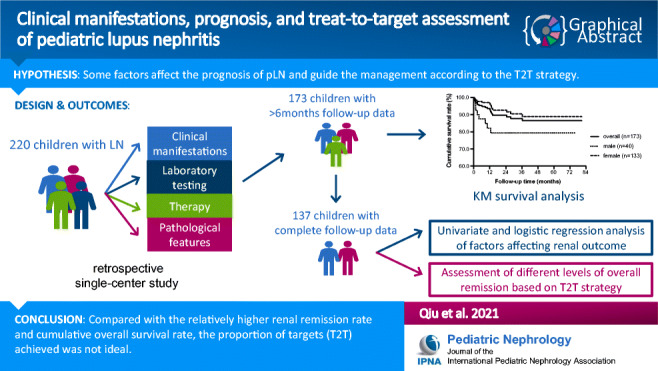
A higher resolution version of the Graphical abstract is available as Supplementary information.

**Supplementary Information:**

The online version contains supplementary material available at 10.1007/s00467-021-05164-y.

## Introduction

Systemic lupus erythematosus (SLE) is a chronic autoimmune disease, and approximately 20% of cases occur in childhood [1]. The disease manifestations of pediatric SLE (pSLE) are often more serious, especially for multiple organ functions such as those of the renal, nervous, and circulatory systems [2]. As one of the most common complications of SLE, lupus nephritis (LN) is also one of the most common causes of secondary glomerulonephritis affecting the long-term prognosis of children. LN has various clinical manifestations and pathological types in children, varying from mild hematuria or proteinuria to nephrotic syndrome and kidney failure, which brings difficulties in clinical diagnosis and treatment of pLN. In addition, the high disease activity of SLE and the toxicity related to long-term use of corticosteroids and immunosuppressive agents cause serious damage to the body. Wu et al. showed that infection and neuropsychiatric lupus (NPL) became the main causes of adult death in SLE [3]. Therefore, treatment in LN should not only focus on renal remission but also aim at controlling systemic disease activity, preventing organ damage, and minimizing comorbidities and drug toxicity. To control disease activity, improve health-related quality of life (HRQoL), and reduce morbidity and mortality, the treat-to-target (T2T) strategy has been proposed in the treatment of SLE since 2014 by the multidisciplinary international task force [4]. Consensus treatment target definitions have been developed and investigated in several adult SLE cohorts [5, 6]. Several possible targets of the T2T strategy include varying levels of remission and low disease activity state (LDAS). Remission would be the main target, but LDAS could be an acceptable alternative when remission is not achievable. Over the last few years, the T2T strategy has appeared to be effective for preventing damage and improving survival in several cohort studies of adult SLE. However, the T2T strategy has not been applied in the field of pediatrics.

Here, we retrospectively analyzed and conducted prognostic follow-up on the clinical data of 220 children with LN. We summarized their clinical manifestations and renal pathological characteristics, and explored the associated factors for prognosis, to improve the prognosis for future cases of LN. Besides, referring to the proposed targets of adult SLE, we evaluated overall remission in these children to understand their current prognosis and help manage pSLE based on the T2T strategy.

## Patients and methods

### Patients

Patients in this study were 220 children who were hospitalized at the Children’s Hospital of Chongqing Medical University, Chongqing, China, from January 2012 to December 2018. All were diagnosed with SLE and LN at ≤18 years of age. The criteria of SLE were based on the American College of Rheumatology (ACR) classification (2012) [7]. LN was diagnosed according to the following criteria [8]: proteinuria > 150 mg/24h, or qualitative urine protein positive for three times per week, or urine protein/urinary creatinine > 0.2 mg/mg, or abnormal urine microalbumin for three times per week; hematuria > 5 RBC/hpf; abnormal function of glomerulus and (or) renal tubules; abnormal kidney biopsy consistent with pathological changes of LN. Kidney biopsy was most highly recommended in LN children with these following characteristics: increasing serum creatinine without compelling alternative causes; proteinuria of ≥ 1.0 g/24h; proteinuria ≥ 0.5 g/24h plus hematuria, defined as ≥ 5 RBC/hpf; proteinuria ≥ 0.5 g/24h plus cellular casts.

**Fig. 1:**
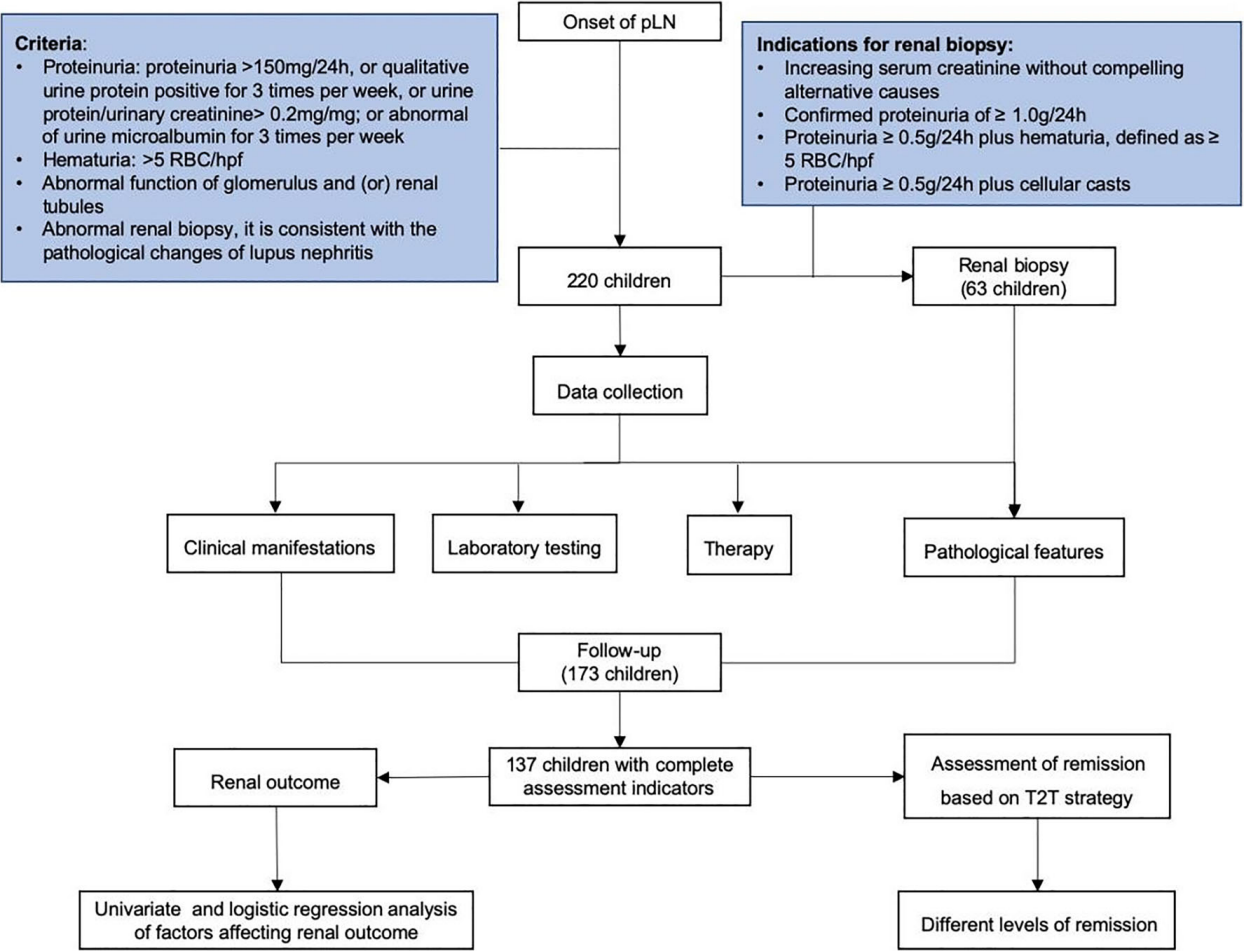
The study design

The study design is shown in Fig. 1.

### Clinical data collection

Clinical data of children with LN were collected retrospectively, including main clinical manifestations and laboratory results. The estimated glomerular filtration rate (eGFR) was calculated using the Schwartz formula [9]. We scored SLE activity per the Systemic Lupus Erythematosus Disease Activity Index 2000 (SLEDAI-2K) [10]. LN was pathologically classified according to the International Society of Nephrology/Renal Pathology Society (ISN/RPS) classification (2003) [11]. The activity index (AI) and chronicity index (CI) of each patient were scored per the semi-quantitative scoring method of the National Institutes of Health (NIH) [12].

### Definition

The definitions of the main clinical manifestations, renal relapse, kidney insufficiency, kidney failure, and treatment non-compliance are shown in Table 1. According to the response criteria of ACR [13], we defined four categories of renal outcomes; the details are also shown in Table 1. Based on the T2T strategy [14], the definitions of outcomes of SLE are shown in Table 2.

**Table 1:** Definitions of clinical manifestations and renal outcomes of SLE

Item	Definition
Manifestation	
Fever	≥38°C (except for fever caused by infection)
Hypertension	The systolic blood pressure and (or) diastolic blood pressure are greater than or equal to the 95th percentile of the blood pressure of children of the same sex, age, and height
Leukopenia	Peripheral blood white blood cell count <4×10^9^/L
Thrombocytopenia	Peripheral blood platelet count <100×10^9^/L
Anemia	Peripheral blood hemoglobin <110g/L
Liver function damage	Doubling of alanine aminotransferase and (or) aspartate aminotransferase compared with the reference value
Hypoproteinemia	Serum albumin<25g/L
Oliguria	Daily urination volume of school-age children is <400mL/m^2^, preschool children is <300mL/m^2^
Nephrotic proteinuria	Proteinuria ≥50mg/kg/day
Non-nephrotic proteinuria	Proteinuria between 0.15g/day and 50mg/kg/day
Renal relapse	Includes a proteinuric relapse (a persistent urine protein >0.15g/day after a complete remission or a doubling of proteinuria which is more than 1g/day after a partial remission) and a nephritic relapse (>25% elevation in serum creatinine associated with increased urinary sediment).
Renal insufficiency	serum creatinine >1.2 mg/dL and creatinine clearance lower than 75 mL/min
CKD stage 5	eGFR<15mL/min/1.73m^2^ for 3 months or entering maintenance dialysis
Treatment non-compliance	failure to follow the doctor’s prescription or stop the medication during treatment, or fail to be hospitalized for regular treatment according to the plan
Renal outcomes of LN	
Complete remission (CR)	(1) eGFR of ≥90 mL/min/1.73 m^2^; (2) proteinuria is <0.15g/day or uPCR is <0.2; (3) inactive sediment (defined as ≤5 RBCs/hpf, ≤5 WBCs/hpf and no cellular casts)
Partial remission (PR)	(1) eGFR of <90 mL/min/1.73 m^2^, 25% increase if baseline eGFR is abnormal, or stable value if normal at baseline; (2) at least 50% reduction in urinary protein, uPCR is 0.2–2.0
No remission (NR)	Stable values for the eGFR or urinary protein
Deterioration	(1) 25% decline in estimated GFR or ESRD; 100% increase in the uPCR; (2) death

*eGFR*, estimated glomerular filtration rate; *hpf*, high-power field; *RBC*, red blood cell; *WBC*, white blood cell; *uPCR*, urinary protein: creatinine ratio; *ESRD*, end-stage renal disease

These children were divided into two groups by renal outcome at the end of follow-up: a remission group including CR and PR, and a failure group including NR and deterioration

**Table 2:** Definition of proposed targets (T2T) in SLE

	Complete remission off therapy	Complete remission on therapy	Clinical remission off therapy	Clinical remission on therapy	LDAS
Clinical activity	No	No	No	No	Yes, but global SLEDAI≤4
Serological activity	No	No	Yes	Yes	Yes, but global SLEDAI≤4
Prednisone	No	≤5mg/day	No	≤5mg/day	≤7.5mg/day
Immunosuppressive drugs	No	Yes	No	Yes	Yes
Antimalarials	Yes	Yes	Yes	Yes	Yes

*LDAS*, low disease activity status

Remission included complete remission off therapy, complete remission on therapy, clinical remission off therapy, and clinical remission on therapy

### Statistical analysis

We statistically analyzed all data using SPSS software version 25.0 (IBM Corp., Armonk, NY, USA). Continuous variables are presented as mean with standard deviation (mean ± SD) or median with interquartile range (IQR). Categorical variables are presented as frequencies and percentages (n (%)). We used Student’s *t-*test, analysis of variance (ANOVA), the Mann-Whitney *U* test, and the Kruskal-Wallis *H* test for comparing continuous variables. Categorical variables were analyzed using the chi-square or Fisher’s exact test. Multifactorial analysis of influencing factors was performed using logistic regression analysis. Kaplan-Meier (KM) analysis was used for survival analysis. Comparisons were performed between groups using the log-rank test. Data were examined at the 95% confidence level, and P value < 0.05 was considered statistically significant. Figures were created with GraphPad Prism 8.

## Results

### Clinical and laboratory characteristics

Of the 220 children with LN included in this study, 50 were males (22.73%) and 170 were females (77.27%), with a male:female ratio of 1:3.4. The mean age at diagnosis was 10.94 ± 2.49 years (minimum, 3.25 years; maximum, 17.00 years). Median disease duration from onset to diagnosis was 1.00 month (IQR 0.40–2.00 months; minimum, 1 day; maximum, 2 years). The main clinical manifestations and laboratory indicators of children with LN at diagnosis are shown in Supplementary Tables 1, 2, and 3. The most common manifestation was proteinuria (81.36%). The median proteinuria was 1.13 g/day (IQR 0.33–2.41g/day). Among those children with proteinuria, 80 (44.69%, 80/179) had nephrotic-range proteinuria. Kidney insufficiency was observed in 51 (23.18%) children at diagnosis. Of initial non-renal manifestations, fever (65.45%) and rash (60.46%) were the most common. Females presented more often with rash (P <0.001) and alopecia (P = 0.026) than males. Antinuclear antibodies (ANA) were detected in nearly all patients (99.09%), followed by anti-double-stranded DNA (anti-dsDNA) antibodies (81.36%) and anti-single-stranded DNA (anti-ssDNA) antibodies (78.18%).

### Pathological characteristics

In this study, a total of 65 children met the indications and willingly underwent kidney biopsy. Two cases were not classified due to limited materials. The distribution and percentage of pathological types in this group are shown in Supplementary Fig. 1. Class IV LN was the most common (21 cases, 33.33%). There are 27 cases (42.86%) of class V LN, including 10 cases of pure class V. No class VI LN was found among the children in this study.

We compared the main laboratory indices at diagnosis of children with class III LN (including class III+V), class IV LN (including class IV+V), and pure class V LN. As shown in Supplementary Table 4, we found significant differences in quantitative levels of 24-h urine proteins (24hUPs), eGFRs, and AI values among the three groups of children. After multiple comparisons of post hoc tests, quantitative levels of 24hUPs were significantly higher in children with class IV LN than in those with class III LN (*Z* = 2.579; P = 0.010), while no significant difference was found between class III LN and pure class V LN children or between class IV LN and pure class V LN children (Supplementary Fig. 2a). The eGFR of class III LN and class IV LN was significantly lower than those of pure class V LN (P = 0.016, P < 0.001, respectively), while no significant difference in eGFR was found between class III LN and class IV LN children (Supplementary Fig. 2b). The AI of class IV LN children was significantly higher than that of class III LN children (P <0.001).

### Initial therapy

As a retrospective case analysis, the treatment strategies were based on the ACR guidelines [15], and no specific treatment strategies were set for this study. In initial therapy, all children in the cohort were treated with an induction regimen of corticosteroids or corticosteroids combined immunosuppressants, primarily cyclophosphamide (CYC), or mycophenolate (MMF), and hydroxychloroquine as the adjunctive treatment. No patients received a combination of CYC and MMF in their initial treatment. Based on the retrospective collection of data, the children were grouped according to whether they received CYC or MMF as induction treatment to compare the efficacy of CYC and MMF. We compared the main indicators at the beginning of the initial therapy, the end of the initial therapy, and the end of follow-up in the two groups. There was no significant difference in 24hUP, serum creatinine (SCr), complement (C)3, C4, anti-dsDNA antibodies, SLEDAI-2K score, or the rate of remission and LDAS between the CYC and MMF groups (Table 3).

**Table 3:** Laboratory parameters in CYC and MMF groups for LN initial therapy

Parameters	CYC (n=104)	MMF (n=20)	P value
Baseline			
24hUP (g/day)	1.77 (0.82–2.97)	1.47 (1.02–2.95)	0.597
Scr (μmoI/L)	56.00 (40.70–94.65)	45.50 (38.00–69.05)	0.354
C3 (g/L)	0.28 (0.17–0.42)	0.29 (0.19–0.47)	0.773
C4 (g/L)	0.05 (0.02–0.08)	0.07 (0.02–0.12)	0.247
Anti-dsDNA (+)	84 (80.77)	15 (75.00)	0.556
SLEDAI-2K	15.16±4.63	13.80±6.83	0.401
End of initial therapy			
24hUP (g/day)	0.98 (0.69–1.60)	0.91 (0.32–1.57)	0.197
SCr (μmoI/L)	49.30 (41.00–58.25)	43.05 (36.10–52.50)	0.100
C3 (g/L)	0.76 (0.55–0.92)	0.74 (0.59–0.88)	0.758
C4 (g/L)	0.16 (0.11–0.19)	0.18 (0.09–0.21)	0.695
Anti-dsDNA (+)	51 (49.04)	11 (55.00)	0.625
SLEDAI-2K	5.97±3.88	6.90±3.46	0.321
Renal remission ^a^	79 (75.96)	13 (65.00)	0.305
End of follow-up			
24hUP (g/day)	0.69 (0.28–1.90)	0.33 (0.19–1.30)	0.180
SCr (μmoI/L)	47.85 (39.35–55.95)	44.50 (38.00–55.00)	0.482
C3 (g/L)	0.74 (0.63–0.89)	0.68 (0.45–0.81)	0.153
C4 (g/L)	0.16 (0.11–0.21)	0.13 (0.08–0.18)	0.195
Anti-dsDNA (+)	35 (33.65)	9 (45.00)	0.331
SLEDAI-2K	5.10±4.71	6.05±5.24	0.417
Renal remission ^a^	73 (70.19)	12 (60.00)	0.369
Overall remission ^b^	41 (39.42)	5 (25.00)	0.221
LDAS (excluding remission)	9 (8.66)	3 (15.00)	0.408

*24hUP*, 24-h urinary protein; *SCr*, serum creatinine; *C3*, complement 3; *C4*, complement 4; *anti-dsDNA*, anti-double-stranded DNA; *SLEDAI-2K*, systemic lupus erythematosus disease activity index 2000; *LDAS*, low disease activity status

^a^Including complete remission and partial remission of renal outcomes

^b^Including complete remission off/on therapy and clinical remission off/on therapy

### Follow-ups and prognosis

As of the follow-up endpoint (December 2019), 173 out of 220 LN children had been followed up and 47 cases were lost to follow-up. The overall follow-up rate of our patients was 78.64% (173/220). The median follow-up duration was 27.20 months (IQR, 15.78–44.45 months), with the shortest 6 months and the longest 81 months.

**Fig. 2:**
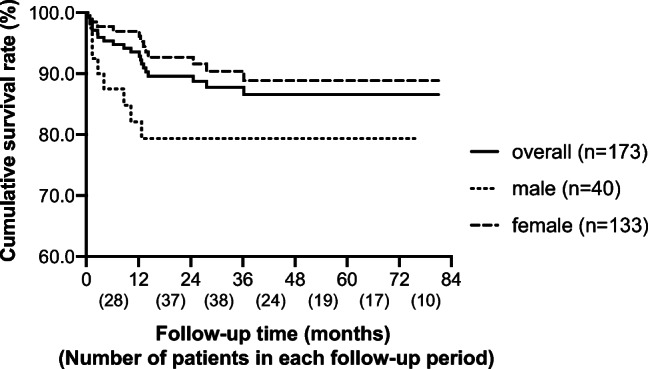
Survival analysis of LN

Death was used as the end event of the survival status. A total of 20 children died during this study, including 8 males and 12 females, with a mortality rate of 11.56% (20/173). According to KM analysis, 12-month (1-year), 36-month (3-year), and 60-month (5-year) cumulative overall survival (OS) rates were 93.5%, 87.8%, and 86.5%, respectively (Fig. 2). The cumulative survival rate of males was lower than that of females, showing a significant difference in log-rank analysis (P = 0.037). Analysis of causes of death in the 20 deceased children showed that infection (10 cases, 50%) was the leading cause of death in all cases, followed by cerebral hemorrhage (4 cases, 20.00%), cerebral hernia (2 cases, 10.00%), interstitial lung disease (2 cases, 10.00%), uremia (1 case, 5.00%), and lupus cardiomyopathy (1 case, 5.00%).

### Renal outcomes

**Fig. 3:**
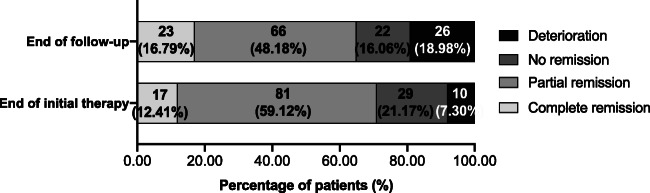
Outcome of 137 LN children at the end of initial therapy and the end of follow-up

Among the 173 children we followed up, 137 children had complete assessment indicators for the renal outcomes (Table 1), whose clinical manifestations were not significantly different from those of the overall population (Supplementary Table 1). The renal outcomes of the 137 children were as follows (Fig. 3). We observed complete remission in 17 cases (12.41%), partial remission in 81 cases (59.12%), no remission in 29 cases (21.17%), and deterioration in 10 cases (7.30%) at the end of the initial therapy, with a renal remission rate of 71.53%. At the same time, 23 children had renal relapses after complete or partial remission, with a renal relapse rate of 23.47% (23/98). At the end of follow-up, we observed complete remission in 23 cases (16.79%), partial remission in 66 cases (48.18%), no remission in 22 cases (16.06%), and deterioration in 26 cases (18.98%), with a total remission rate of 64.96%. At the end of follow-up, two children were observed with chronic kidney disease (CKD) stage 5 independently at the 18th and 47th months of disease duration. KM analysis showed that the 5-year cumulative renal survival rate was 97.1%.

### Risk factors for poor prognosis of renal outcomes

We divided these 137 children into two groups by renal outcomes at the end of follow-up: a remission group including CR and PR, and a failure group including NR and deterioration. We performed univariate analysis to compare demographic data, clinical manifestations, main laboratory indicators, and therapy between the two groups to analyze the risk factors leading to poor prognosis of kidney injury. The results showed that hypoalbuminemia, anti-dsDNA positivity, nephrotic-range proteinuria, hypertension, nervous-system involvement, SLEDAI-2K score, SCr, eGFR, and treatment compliance were significantly different between the two groups of children with LN (P <0.05). We found significant differences in treatment plan during the induction period and in treatment compliance between the two groups of children with LN (P <0.05). Further multiple-comparison results showed that the remission rate of children receiving CYC was significantly higher than that of children receiving corticosteroid treatment alone (*χ*^2^ = 7.982; P = 0.010). No significant difference in induction remission rate was found between children in the CYC and the MMF groups (*χ*^2^ = 0.808; P = 0.369). Univariate analysis of renal pathological indicators in LN children undergoing kidney biopsy showed a significant difference in AI between the two groups (*Z* = 2.445; P = 0.014) (Supplementary Table 5).

### Multivariate regression analysis of factors for poor prognosis of renal outcomes

Factors with P < 0.05 in univariate analysis, including hypoalbuminemia, higher SCr at diagnosis, lower eGFR at diagnosis, anti-dsDNA positivity, heavy proteinuria, hypertension, nervous-system involvement, treatment non-compliance, and SLEDAI-2K score, were included in the logistic regression analysis (Table 4). The results showed that hypertension, nervous-system involvement, treatment non-compliance, and lower eGFR at diagnosis were independent risk factors affecting the prognosis of renal outcomes.

**Table 4:** Logistic regression analysis of risk factors leading poor prognosis of 137 LN children

Influencing factors	P value	OR	95%CI
Hypoproteinemia	0.845	0.851	0.169–4.277
SCr at diagnosis	0.163	1.012	0.995–1.029
eGFR at diagnosis	0.021	1.020	1.003–1.037
Anti-dsDNA (+)	0.140	2.567	0.735–8.964
Nephrotic-range proteinuria	0.324	2.272	0.445–11.590
Hypertension	0.011	3.448	1.326–8.970
Nervous-system involvement	0.005	4.240	1.531–11.739
Treatment non-compliance	0.001	6.433	2.228–18.578
SLEDAI-2K	0.560	0.974	0.892–1.064

*SCr*, serum creatinine; *eGFR*, estimated glomerular filtration rate; *anti-dsDNA*, anti-double-stranded DNA; *SLEDAI-2K*, Systemic Lupus Erythematosus Disease Activity Index 2000

### Assessment of overall treatment targets

Based on the T2T strategy, this study evaluated the conditions of 137 children at the end of follow-up (Table 5). We found that 47 children (34.31%) achieved varying levels of remission. Of these, 5 (3.65%) achieved complete remission off therapy, 19 (13.87%) achieved complete remission on therapy, 1 (0.73%) had clinical remission off therapy, and 22 (16.06%) had clinical remission on therapy. Twelve (8.76%) achieved LDAS. Seventy-eight (56.93%) of these children did not achieve any level of remission or LDAS.

**Table 5:** Various types of remission at end of follow-up in 137 LN children

Targets	n	Percentage (%)
Remission	47	34.31
Complete remission off therapy	5	3.65
Complete remission on therapy	19	13.87
Clinical remission off therapy	1	0.73
Clinical remission on therapy	22	16.06
LDAS (excluding remission)	12	8.76

*LDAS*, low disease activity status

## Discussion

PSLE is an autoimmune disease that causes multi-system damage and LN is one of its most important complications. Childhood LN is often more serious than LN onset in later adulthood [16]. Therefore, early diagnosis, timely treatment, and reasonable management are essential to improving the prognosis of children with LN.

In our study, the epidemiological characteristics and clinical manifestations were similar to previous reports. The initial manifestation of kidney involvement was mainly proteinuria. Of initial non-renal manifestations, fever and rash were the most common. Compared with adult patients, symptoms such as oral ulcers and alopecia in pediatric patients were relatively rare. Also, females presented more often with rash and alopecia than males in our study. Compared with females, males often have atypical clinical manifestations and heavily rely on examinations for diagnosis.

Few children with LN in our center had neuropsychiatric symptoms. However, multivariate regression analysis showed that nervous-system involvement was an independent risk factor affecting prognosis in pSLE, and was also the second-leading cause of death in this study (cerebral hemorrhage and cerebral hernia). Children with NPL are sometimes ignored in the early stage due to mild or asymptomatic clinical presentation, leading them to be misdiagnosed or undiagnosed. However, once severe symptoms such as disturbance of consciousness, epilepsy, and brain herniation occur, the disability and fatality rates of these patients are relatively high. Therefore, it is necessary to be vigilant in clinical practice. Electroencephalography, cerebrospinal fluid tests, head imaging, and other auxiliary examinations should be used to comprehensively evaluate children with SLE.

Kidney biopsy data in this study showed that class IV LN was the most common pathological type. These findings were consistent with those of another study [17]. Clinical manifestations of LN also had a certain relationship to the pathological type of LN. For example, urine protein level was significantly higher in class IV LN than in class III LN, and kidney function was better in pure class V LN than in proliferative LN. However, the clinical manifestations of children with LN were not completely consistent with the pathological types of LN. Biopsies of some children with mild proteinuria confirmed them to have class IV LN, with relatively high AI and CI scores. In the course of disease progression, the pathological type of LN can also change. Repeat kidney biopsy is also recommended in some cases, such as worsening or refractoriness to treatment. In this study, the data showed that one patient underwent kidney biopsy again after an interval of 2 years, and the patient’s pathological type changed from class V LN to class III+V LN, with CI score reaching 5 points. So when children with SLE have evidence of kidney damage, a timely kidney biopsy is recommended to clarify the pathological type and provide reasonable treatment in time.

Consistent with the previous study by Smith et al. [18], regarding regimens for initial therapy, no significant differences in renal remission were found between the CYC and MMF groups at the end of the initial therapy. Notably, treatment non-compliance was an independent risk factor affecting the prognosis of LN children. During follow-up and while communicating with parents of LN children, we learned that some children failed to follow medical advice for treatment due to insufficient understanding of the disease or emotional disorders. HRQoL has attracted more and more attention and is usually overlooked in the management of SLE. Donnelly et al. showed that emotional symptoms such as anxiety and depression predict the decline of HRQoL in children with SLE [19]. Therefore, in clinical treatment, we must provide not only health education for the parents of these children but also appropriate psychological counseling for the children themselves, strengthen their understanding of the disease, and treat their psychiatric disorders.

Vachvanichsanong et al. reported that sepsis was the leading cause of death in children with LN in Thailand [20]. As in most reports, infection was also the primary cause of death in children with LN in this study (50.00%). Therefore, for children with infections, clinical pathogenic examinations should be completed in a timely fashion, and antibiotics should be used rationally to control the infections.

This study showed that at the end of the follow-up, the renal remission rate (64.97%) and cumulative overall survival rate (86.50%, 5-year) were relatively higher. However, assessing the achievement of targets with T2T strategy, only 34.31% of children achieved varying levels of remission, and 8.76% of children achieved LDAS. Compared with studies of adult SLE [21, 22], these proportions were lower. Franklyn et al. reported that 88.5% of 191 patients in their cohort achieved LDAS at least once, and the cumulative duration of LDAS was 41% of the total period of observation [14]. Compared with adults, children are more active in SLE and receive more intensive immunosuppressive therapy. Even if the disease is in remission, it is mostly clinical remission on therapy, meaning that long-term use of steroids and immunosuppressants is toxic and accumulates more organ damage over time. Therefore, pediatric nephrologists also need to pay attention to the systemic damage of SLE in the treatment of pLN and try to use the T2T strategy to guide the management of these children. It is necessary to consider other optimized treatment programs to improve the prognosis of children with LN. Emerging therapeutic antibodies have become an alternative and have been extensively studied for the treatment of SLE, such as rituximab and belimumab [23, 24]. T2T is currently the most advocated strategy in the management of SLE. However, as yet there is a lack of long-term observation of organ damage and outcomes based on the T2T strategy in children. The specific targets of pSLE have not yet been defined. A recent study [25] of pSLE parents by interviews showed that the concept of T2T was acceptable to families. Most families were reluctant to tolerate ongoing symptoms, and remission was a more attractive treatment target for them than LDAS. Therefore, this requires further large-sample, multi-center, and even prospective studies to develop the definition of remission that would be acceptable to families. The precise minimum doses of immunosuppressive drugs and corticosteroids allowed should also be defined for remission or LDAS.

Several limitations of this study should be mentioned. We retrospectively collected and analyzed data in a single center for pSLE patients with kidney involvement. We have identified several risk factors that affect the renal prognosis, which is only part of the risk factors for poor prognosis of pSLE since treatment of SLE is the integrated management of multiple organ injuries. We also pay attention to the difference between renal prognosis and the targets of T2T. However, due to the relatively small samples from a single-center study, it is difficult to conduct statistical analysis based on T2T target groupings. Besides, as a retrospective study, we only assessed the overall remission of children with LN at the end of the follow-up, so we could not accurately confirm the time to achieve each target and the duration of remission without prospective application of the T2T strategy in the management of pLN in this cohort. Our team plans to continue this study and incorporate multi-center data, which would hopefully find more predictors for poor prognosis of pSLE, different remissions, and LDAS and confirm the definitions of T2T in pSLE.

In conclusion, this is one of the largest cohort studies of pLN from a single center in China. We summarized the clinical manifestations and analyzed the associated factors for the prognosis of pLN. Further to this, the innovation of this study is that we used the promising T2T strategy to assess the outcomes of these children. Compared with the relatively higher renal remission rate and cumulative overall survival rate, the proportion of targets (T2T) achieved was not ideal. This suggested that we also need to pay attention to the practice of T2T for pSLE.

## Declarations

Graphical abstract (PPTX 77.3 kb) ESM 1 (DOCX 83 kb)
