# Supplementary material for: Clinical manifestations, prognosis, and treat-to-target assessment of pediatric lupus nephritis
Source: Pediatr Nephrol. 2021 Aug 11;37(2):367–76. doi: 10.1007/s00467-021-05164-y (PMC8816762; doi:10.1007/s00467-021-05164-y)
Supplement: Graphical abstract — (PPTX 77.3 kb) [file 467_2021_5164_MOESM1_ESM.pptx]

## Slide 1
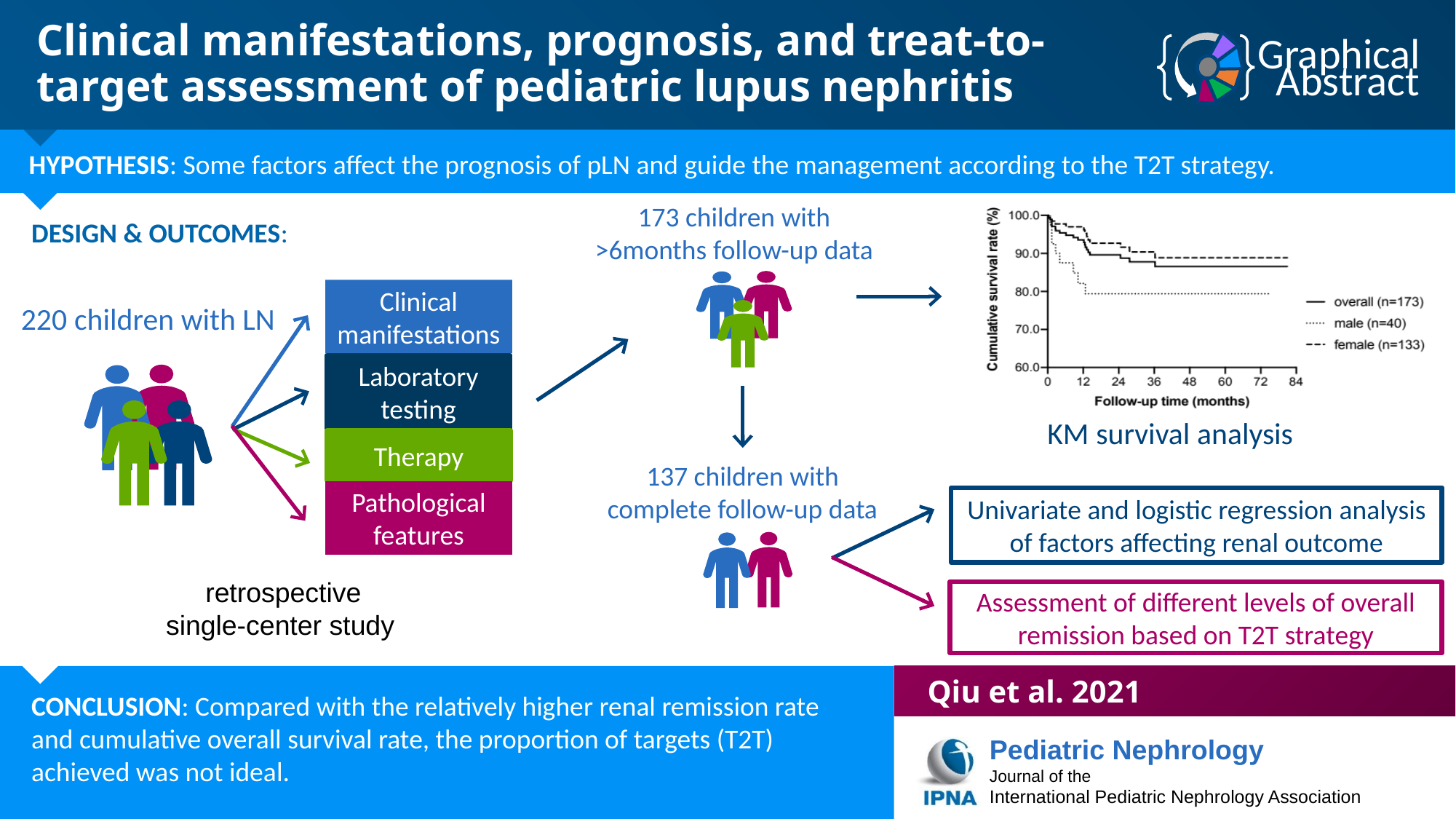

Clinical manifestations, prognosis, and treat-to-target assessment of pediatric lupus nephritis
HYPOTHESIS: Some factors affect the prognosis of pLN and guide the management according to the T2T strategy.
173 children with >6months follow-up data
DESIGN & OUTCOMES:
Clinical manifestations
220 children with LN
Laboratory testing
KM survival analysis
Therapy
137 children with complete follow-up data
Pathological features
Univariate and logistic regression analysis of factors affecting renal outcome
retrospective
single-center study
Assessment of different levels of overall remission based on T2T strategy
Qiu et al. 2021
CONCLUSION: Compared with the relatively higher renal remission rate and cumulative overall survival rate, the proportion of targets (T2T) achieved was not ideal.
