# Supplementary material for: Clinical manifestations, prognosis, and treat-to-target assessment of pediatric lupus nephritis
Source: Pediatr Nephrol. 2021 Aug 11;37(2):367–76. doi: 10.1007/s00467-021-05164-y (PMC8816762; doi:10.1007/s00467-021-05164-y)
Supplement: ESM 1 — (DOCX 83 kb) [file 467_2021_5164_MOESM2_ESM.docx]

**Supplementary Tables**

**Table 1** Comparison of clinical manifestations at diagnosis in different follow-up groups

| Item | Overall  (n=220) | Children with any follow-up data  (n=173) | Children with complete follow-up data  (n=137) | P value |
| --- | --- | --- | --- | --- |
| Constitutional |  |  |  |  |
| Fever | 144 (65.45) | 117(67.63) | 99 (72.26) | 0.399 |
| Hematologic |  |  |  |  |
| Anemia | 95 (43.18) | 71 (41.04) | 63 (45.99) | 0.690 |
| Leukopenia | 87 (39.55) | 55 (31.79) | 46 (33.58) | 0.247 |
| Thrombocytopenia | 64 (29.09) | 42 (24.28) | 29 (21.17) | 0.230 |
| Neuropsychiatric |  |  |  |  |
| Headache | 18 (8.18) | 11 (6.36) | 8 (5.84) | 0.694 |
| Seizure | 14 (6.36) | 6 (3.47) | 3 (2.19) | 0.168 |
| Mucocutaneous |  |  |  |  |
| Rash | 133 (60.45) | 108 (62.43) | 92 (67.15) | 0.443 |
| Oral ulcers | 18 (8.18) | 14 (8.09) | 10 (7.30) | 0.958 |
| Alopecia | 16 (7.27) | 11 (6.36) | 9 (6.57) | 0.975 |
| Serosal |  |  |  |  |
| Pericardial effusion | 72 (32.72) | 55 (31.79) | 43 (31.39) | 0.971 |
| Pleural effusion | 46 (20.91) | 32 (18.50) | 24 (17.52) | 0.720 |
| Musculoskeletal |  |  |  |  |
| joint pain | 78 (35.45) | 54 (31.21) | 46 (33.58) | 0.674 |
| arthritis | 32 (14.55) | 26 (15.03) | 19 (13.87) | 0.975 |
| Renal |  |  |  |  |
| Proteinuria | 179 (81.36) | 135 (78.03) | 111 (81.02) | 0.690 |
| Hematuria | 151 (68.64) | 126 (72.83) | 105 (76.64) | 0.259 |
| Edema | 85 (38.64) | 55 (31.79) | 42 (30.66) | 0.216 |
| Renal insufficiency | 51 (23.18) | 37 (21.39) | 32 (23.36) | 0.897 |
| Oliguria | 48 (21.82) | 33 (19.08) | 25 (18.25) | 0.678 |

**Table 2** Comparison of clinical manifestations in male and female

| Item | Overall  (n=220) | Male  (n=50) | Female  (n=170) | P value |
| --- | --- | --- | --- | --- |
| Constitutional |  |  |  |  |
| Fever | 144 (65.45) | 32(64.00) | 112 (65.88) | 0.806 |
| Hematologic |  |  |  |  |
| Anemia | 95 (43.18) | 19 (38.00) | 76 (44.71) | 0.400 |
| Leukopenia | 87 (39.55) | 15 (30.00) | 72 (42.35) | 0.116 |
| Thrombocytopenia | 64 (29.09) | 15 (30.00) | 49 (28.82) | 0.872 |
| Neuropsychiatric |  |  |  |  |
| Headache | 18 (8.18) | 6 (12.00) | 12 (7.06) | 0.253 |
| Seizure | 14 (6.36) | 4 (8.00) | 10 (5.88) | 0.527 |
| Mucocutaneous |  |  |  |  |
| Rash | 133 (60.45) | 17 (34.00) | 116 (68.24) | <0.001 |
| Oral ulcers | 18 (8.18) | 2 (4.00) | 16 (9.41) | 0.377 |
| Alopecia | 16 (7.27) | 0 (0.00) | 16 (9.41) | 0.026 |
| Serosal |  |  |  |  |
| Pericardial effusion | 72 (32.72) | 13 (26.00) | 59 (34.71) | 0.249 |
| Pleural effusion | 46 (20.91) | 11 (22.00) | 35 (20.59) | 0.829 |
| Musculoskeletal |  |  |  |  |
| joint pain | 78 (35.45) | 15 (30.00) | 63 (37.06) | 0.359 |
| arthritis | 32 (14.55) | 6 (12.00) | 26 (15.29) | 0.561 |
| Renal |  |  |  |  |
| Proteinuria | 179 (81.36) | 44 (88.00) | 135 (79.41) | 0.170 |
| Hematuria | 151 (68.64) | 35 (70.00) | 116 (68.24) | 0.813 |
| Edema | 85 (38.64) | 23 (46.00) | 62 (36.47) | 0.224 |
| Renal insufficiency | 51 (23.18) | 14 (28.00) | 37 (21.76) | 0.358 |
| Oliguria | 48 (21.82) | 14 (28.00) | 34 (20.00) | 0.229 |

**Table 3** Laboratory parameters at diagnosis of whole cohort

| Parameters | Mean±SD | Normal range |
| --- | --- | --- |
| WBC (×10^9^/L) | 6.32±4.76 | 4-12 |
| Platelet (×10^9^/L) | 162.09±102.31 | 100-380 |
| Hemoglobin (g/L) | 92.79±18.90 | 110-160 |
| BUN (mmol/L) | 6.35 (3.90-9.31) | 2.2-7.14 |
| SCr (μmoI/L) | 56.10 (42.65-93.65) | 14-60 |
| eGFR (mL/min/1.73m^2^) | 119.28±47.85 | >90 |
| 24hUP (g/day) | 1.13 (0.33-2.41) | <0.15 |
| Albumin (g/L) | 29.66±8.12 | 38-55 |
| ALT (U/L) | 30.30 (19.50-51.10) | 0-40 |
| AST (U/L) | 38.00 (25.50-70.90) | 0-45 |
| ESR (mm/h) | 48.78±34.89 | 0-15 |
| C3 (g/L) | 0.28 (0.18-0.43) | 0.7-2.06 |
| C4 (g/L) | 0.05 (0.03-0.09) | 0.11-0.61 |
| Autoantibody positivity |  |  |
| ANA | 218/220 (99.09%) | - |
| anti-dsDNA | 179/220 (81.36%) | - |
| anti-ssDNA | 172/220 (78.18%) | - |
| AnuA | 158/220 (71.82%) | - |
| anti-P | 109/220 (49.55%) | - |
| anti-SSA antibodies | 98/220 (44.55%) | - |
| anti-Sm antibodies | 76/220 (34.55%) | - |
| anti-SSB antibodies | 43/220 (19.55%) | - |
| ACA | 22/92 (23.91%) | - |
| ANCA | 18/160 (11.25%) | - |

*WBC*, white blood cells; *BUN*, blood urea nitrogen; *SCr*, serum creatinine; *eGFR*, estimated glomerular filtration rate; *24hUP*, 24-hour urinary protein; *ALT*, alanine aminotransferase; *AST*, aspartate aminotransferase; *ESR*, erythrocyte sedimentation rate; *C3*, complement 3; *C4*, complement 4; *ANA*, antinuclear antibodies; *anti-dsDNA*, anti-double-stranded DNA; *anti-ssDNA*, anti-single-stranded DNA; *AnuA*, anti-nucleosome antibody; *anti-P*, Anti-ribosomal-P antibodies; *SSA*, Sjögren's syndrome A; *SSB*, Sjögren's syndrome B; *ACA*, anti-cardiolipin antibody; *ANCA*, anti-neutrophil cytoplasm antibodies

**Table 4** Laboratory parameters of various pathological types of LN at diagnosis

| Parameters | Pathological types | | | P value |
| --- | --- | --- | --- | --- |
|  | Class III (III+V)  (n=16) | Class IV (IV+V)  (n=34) | Pure class V  (n=10) |  |
| Albumin (g/L) | 29.05±6.69 | 25.33±4.76 | 25.20±8.23 | 0.107 |
| 24hUP (g/day) | 1.60 (0.67-2.43) | 2.21 (1.64-2.89) | 2.07 (1.08-3.95) | 0.041 |
| SCr (μmoI/L) | 66.70 (46.25-99.23) | 66.50 (46.75-110.38) | 49.20 (36.75-50.38) | 0.063 |
| eGFR (mL/min/1.73m^2^) | 106.56±52.80 | 107.40±47.90 | 150.93±15.33 | 0.028 |
| C3 (g/L) | 0.33 (0.22-0.42) | 0.28 (0.22-0.37) | 0.28 (0.22-0.46) | 0.791 |
| C4 (g/L) | 0.07 (0.03-0.11) | 0.06 (0.03-0.08) | 0.05 (0.04-0.10) | 0.833 |
| AI | 7.00 (6.00-9.75) | 13.00 (10.00-15.00) | - | <0.001 |
| CI | 0.00 (0.00-0.75) | 0.00 (0.00-1.00) | - | 0.134 |
| SLEDAI-2K | 14.13±4.76 | 14.91±4.24 | 12.60±2.84 | 0.310 |

*24hUP*, 24-hour urinary protein; *SCr*, serum creatinine; *eGFR*, estimated glomerular filtration rate; *C3*, complement 3; *C4*, complement 4; *AI,* activity index; *CI,* chronicity index; *SLEDAI-2K,* systemic lupus erythematosus disease activity index 2000

**Table 5** Correlation of demographic features, laboratory parameters, clinical manifestations and treatments between remission group and failure group at end of follow-up

| Influencing factors | Remission group  (n=89) | Failure group  (n=48) | P value |
| --- | --- | --- | --- |
| Sex (male to female) | 19:70 | 16:32 | 0.125 |
| Age at diagnosis (years) | 10.50 ± 2.61 | 10.88 ± 2.22 | 0.394 |
| Course of disease at diagnosis(months) | 0.67 (0.33-2.00) | 1.00 (0.54-2.75) | 0.108 |
| Coombs test positive | 60 (67.42) | 32 (66.67) | 0.929 |
| Elevated transaminase | 13 (14.61) | 12 (25.00) | 0.133 |
| Hypoproteinemia | 31 (34.83) | 26 (54.17) | 0.025 |
| BUN (mmol/L) | 6.32 (4.59 - 9.35) | 7.24 (4.19 - 9.31) | 0.773 |
| SCr (μmoI/L) | 51.00 (40.90-81.40) | 65.2 (47.60 - 109.75) | 0.015 |
| eGFR (mL/min/1.73m^2^) | 125.78 ± 47.91 | 107.22 ± 45.81 | 0.030 |
| anti-dsDNA (+) | 67 (75.28) | 43 (89.58) | 0.045 |
| C3 (g/L) | 0.30 (0.17-0.45) | 0.23 (0.18-0.34) | 0.089 |
| Low serum C3 levels | 68 (76.40) | 41 (85.42) | 0.212 |
| C4 (g/L) | 0.05 (0.03 - 0.09) | 0.04 (0.02 - 0.08) | 0.130 |
| Low serum C4 levels | 61 (68.54) | 37 (77.08) | 0.290 |
| 24hUP (g/day) | 1.26 (0.50-2.46) | 1.60 (0.74 - 3.77) | 0.079 |
| Nephrotic-range proteinuria | 37 (41.57) | 29 (60.42) | 0.035 |
| Hypertension | 28 (31.46) | 25 (52.08) | 0.018 |
| Nervous-system involvement | 14 (15.73) | 20 (41.67) | 0.001 |
| Cardiac involvement | 28 (31.46) | 21 (43.75) | 0.152 |
| SLEDAI-2K | 14.15 ± 5.10 | 16.71 ± 5.23 | 0.006 |
| Regimens for initial therapy |  |  |  |
| Corticosteroids | 4 (4.49%) | 9 (18.75) | 0.018 |
| CYC (combined with corticosteroids) | 73 (82.02) | 31 (64.58) |  |
| MMF (combined with corticosteroids) | 12 (13.48) | 8 (16.67) |  |
| Treatment non-compliance | 11 (12.36) | 19 (39.58) | <0.001 |
| Pathological type |  |  |  |
| III、III+V | 13 (32.50) (n=40) | 2 (12.50) (n=16) | 0.341 |
| IV、IV+V | 20 (50.00) (n=40) | 11 (68.75) (n=16) |  |
| V | 7 (17.50) (n=40) | 3 (18.75) (n=16) |  |
| AI | 9.50 (7.00 - 13.00) | 13.00 (12.00 - 15.00) | 0.014 |
| CI | 0.00 (0.00 - 1.00) | 0.00 (0.00 - 1.00) | 0.812 |
| Glomerular sclerosis | 15 (37.50) (n=40) | 8 (50.00) (n=16) | 0.287 |
| Crescent formation | 17 (42.50) (n=40) | 6 (37.50) (n=16) | 0.486 |
| Atrophy of renal tubules | 7 (17.50) (n=40) | 4 (25.00) (n=16) | 0.384 |
| Interstitial fibrosis | 11 (27.50) (n=40) | 5 (31.25) (n=16) | 0.510 |
| Vascular disease | 5 (12.50) (n=40) | 3 (18.75) (n=16) | 0.412 |

*BUN*, blood urea nitrogen; *SCr*, serum creatinine; *eGFR*, estimated glomerular filtration rate; *anti-dsDNA*, anti-double-stranded DNA; *C3*, complement 3; *C4*, complement 4; *24hUP*, 24-hour urinary protein; *SLEDAI-2K*, systemic lupus erythematosus disease activity index 2000; *CYC*, cyclophosphamide; *MMF*, mycophenolate mofetil; *AI*, activity index; *CI*, chronicity index

**Supplementary Figures**

**Fig. 1** LN pathological types in 63 patients who underwent renal biopsy

**Fig. 2** Laboratory parameters of various pathological types of LN. **a** 24hUPs. **b** eGFRs
